# 薏仁多糖诱导人肺癌A549细胞凋亡

**DOI:** 10.3779/j.issn.1009-3419.2012.11.04

**Published:** 2012-11-20

**Authors:** 相义 卢, 薇 刘, 成 罗

**Affiliations:** 300457 天津，天津科技大学食品工程与生物技术学院 School of Food Engineering and Biotechnology, Key Laboratory of Food Nutrition and Safety of Ministry of Education, Tianjin University of Science and Technology, Tianjin 300457, China

**Keywords:** 薏仁多糖, 肺肿瘤, 细胞凋亡, 半胱氨酰天冬氨酸酶-3, 半胱氨酰天冬氨酸酶-9, Coix polysaccharide, Lung neoplasmsl, Apoptosis, Caspase-3, Caspase-9

## Abstract

**背景与目的:**

薏仁是禾木科植物薏米的干燥成熟种仁，是常用的中药，又是普遍、常吃的食物。本研究旨在探讨薏仁多糖诱导A549细胞凋亡的作用。

**方法:**

本研究通过水提醇沉法提取粗的薏仁多糖，再通过透析、离子交换柱层析等方法得到薏仁多糖组份（CP-1）。采用MTT测定A549细胞的存活率；扫描电镜观察细胞形态；流式细胞仪检测细胞周期；RT-PCR测定*caspase-3*和*caspase-9*两种基因相对表达量；单细胞凝胶电泳检测细胞DNA变化。

**结果:**

MTT测得薏仁多糖对A549细胞存活率有明显的生长抑制作用。扫描电子显微镜观察到测试组A549细胞产生凋亡小体，流式细胞仪分析薏仁多糖处理导致A549细胞S期阻滞，并产生凋亡峰。RT-PCR结果表明与对照组比较，测试组caspase-3和caspase-9表达量上升。通过DNA彗星实验薏仁多糖可延长尾距，表明DNA断裂破碎。

**结论:**

研究结果表明薏仁多糖组分可有效地诱发A549癌细胞凋亡。

## Abstract (en)

**Background and objective:**

Coix seeds are commonly used in Traditional Chinese Medicine and ingested through daily diet. The aim of this study is to analyze the apoptotic effect of coix polysaccharides on A549 cells.

**Methods:**

A fraction of polysaccharides was isolated from coix seeds and extracted by ethanol precipitation. The extract was then purified by dialysis and DEAE-52 ion-exchange chromatography. Cell viability was determined by the MTT assay. Cell morphology was observed by scanning electronic microscopy (SEM), and cell cycle was detected by flow cytometry (FCM). The relative quantities of caspase-3 and caspase-9 were determined by RT-PCR.

**Results:**

Coix polysaccharides exerted remarkable inhibitory effects on A549 cell proliferation. Apoptotic bodies were observed by SEM. Apoptotic induction was also verified by DNA accumulation using propidium iodide nucleus staining in the S phase by flow cytometry, as well as by DNA fragmentation using the comet assay. Regarding the molecular mechanism of apoptosis induction, the gene expression of *caspase-3* and *caspase-9* increased after coix polysaccharide treatment.

**Conclusion:**

Polysaccharide fraction CP-1 induced A549 cell apoptosis.

薏米（Coix）别名薏苡仁、米仁、薏珠子、菩提子、芑实、解蠡等，广泛地种植于亚洲、非洲和地中海周边的温暖地区。薏米的干燥成熟种仁叫做薏仁，薏仁质坚实，断面白色，粉性，气微，味微甜，薏仁通过烘烤或加工成薏米粉食用^[1]^。它含有丰富的蛋白质、脂肪、碳水化合物和多种氨基酸、维生素、无机盐，其中主要的功能性成分是脂肪和糖类。在传统的中药中作为一种利尿剂、抗炎药物、抗癌药物、镇痛剂和一种营养物^[2]^。研究表明薏仁中含有大量脂肪酸包括棕榈酸、硬脂酸、十八碳二烯酸、硬脂酸、油酸、亚油酸等^[3]^，其中含有的寡聚糖具有DPPH自由基清除能力和脂质抗氧化能力^[4]^。薏仁组份已被证实具有降血糖^[5]^，提高免疫力^[6]^等作用。本研究提取纯化得到薏仁多糖（CP-1）并作用于肺癌细胞A549细胞，观察其对肺癌细胞细胞凋亡作用的影响，为以后进一步研究CP-1的抗肿瘤作用提供参考。

## 1 材料与方法

### 1.1 材料与试剂

人非小细胞肺癌细胞A549细胞实验室自有；薏仁购于天津滨海新区TESCO超市；DEAE-52柱材料购于北京索莱宝公司；淀粉酶购于北京索莱宝公司；糖化酶购于北京索莱宝公司；透析袋购于上海欧韦达仪器科技有限公司；胎牛血清购于GIBCO公司；改良型PRMI-1640培养基购于美国Thermo公司；1%青链霉素混合液购于北京索莱宝公司；胰酶购于北京索莱宝公司；二甲基亚砜分析纯购于北京索莱宝公司；噻唑蓝购于北京索莱宝公司；多聚甲醛购于北京索莱宝公司；碘化丙啶购于美国Sigma公司；RNase Inhibitor购于美国MBI公司；总RNA提取试剂盒Qiagen购于德国Hilden公司；逆转录试剂盒Fermentas购于美国Hanover公司；其它试剂为分析纯。

### 1.2 仪器与设备

KW-1000DC恒温水浴锅购自金坛市瑞华仪器有限公司；高速离心机购自美国Thermo公司；SU-1510电子扫描显微镜购自日本日立公司；真空冷冻干燥机购自美国Thermo公司；BT-200B数显恒流泵购自上海沪西分析仪器有限公司；紫外全波长扫描仪购自美国Agilent公司；酶标仪购自美国Thermo公司；EOVS-FL倒置荧光显微镜购自德国AMG公司；Cytomics FC 500流式细胞仪购自美国Beckman Coulter公司；PCR仪购自德国biometra公司；DYY-8C电泳仪购自北京市六一仪器厂；凝胶成像系统购自Bio-RAD公司。

### 1.3 薏仁多糖的提取

#### 1.3.1 薏仁多糖提取工艺流程

薏仁→筛选→粉碎→脱脂→提取→去淀粉→浓缩→醇沉→干燥→薏仁粗多糖。

#### 1.3.2 薏仁多糖提取

挑选籽粒饱满、色泽洁白、无虫蛀、无霉斑的薏仁置于超微粉碎机中，粉碎过筛。使用索式提取器分别用乙酸乙酯和石油醚脱脂，置于三角瓶中，加pH 5.2的蒸馏水于水浴锅中90 ℃提取。提取后趁热离心，取上清液冷却，按20 IU/g加淀粉酶到提取液中水浴温度60 ℃、pH7.0去淀粉直到碘检不变色；接着按18 IU/g加糖化酶，在水浴温度50 ℃-60 ℃，pH6.5条件下处理1 h。100 ℃水浴条件下灭活酶10 min，然后在3, 000 r/min下离心10 min，上清液使用旋转蒸发仪浓缩，加入3倍浓缩液体积的95%冰乙醇沉淀过夜离心，真空冷冻干燥沉淀得薏仁粗多糖。

### 1.4 薏仁多糖的纯化

#### 1.4.1 纯化工艺流程

薏仁粗多糖→脱蛋白→脱色→透析→DEAE-52柱层析→真空冷冻干燥→CP-1。

#### 1.4.2 脱蛋白

将CP-1粉末溶解在蒸馏水中，加入1/3体积的Sevage试剂（氯仿:正丁醇=4:1），剧烈震荡30 min，3, 000 r/min离心15 min，用分液漏斗除去有机相与水相之间的变形蛋白沉淀，水相重复上述操作，至没有变性蛋白沉淀产生。用3倍体积95%冰乙醇沉淀，离心，蒸馏水溶解。

#### 1.4.3 脱色

CP-1水溶液以浓氨水调至pH8.0滴加30%H_2_O_2_至浅黄色，于50 ℃水浴保温2 h。

#### 1.4.4 透析

用截留相对分子质量为8, 000-14, 000的透析袋流水透析2 d。透析后将CP-1浓缩，真空冷冻干燥。

#### 1.4.5 DEAE-52离子交换柱层析

在DEAE-52纤维素层析柱中，加样5 mL以0.1 mol/L NaCl溶液洗脱（恒流泵流速：1.8 r/min-2.1 r/min，5 mL/管）用苯酚硫酸法监测换位检测。将含有CP-1的部分合并，浓缩，真空冷冻干燥得CP-1纯品。苯酚硫酸法测得CP-1的糖含量为85%。

### 1.5 紫外全波长扫描

将CP-1溶解于甲醇中，于200 nm-800 nm区间进行紫外全波长扫描，观察在260 nm、280 nm处是否有吸收峰。

### 1.6 细胞培养

A549细胞培养于含10%胎牛血清和1%青链霉素混合液（100×）的改良型PRMI-1640培养液中，于培养箱（37 ℃、5%CO_2_）中恒温培养，取对数期细胞进行实验。

### 1.7 MTT检测对A549细胞存活率影响

对数期成长的细胞，胰酶消化，培养于96孔板中，加入不同浓度的CP-1（0、10 μg/mL、25 μg/mL、50 μg/mL、75 μg/mL、100 μg/mL、200 μg/mL和300 μg/mL）处理24 h、48 h、72 h之后避光加入5 mg/mL的MTT（噻唑蓝）20 μL 37 ℃培养4 h，3, 000 r/min离心5 min，吸去上清，加入150 μL二甲基亚砜（DMSO）。用微型振荡器振荡混匀，酶标仪在570 nm下测定吸光度值，以DMSO处理的A549细胞作为阴性对照组细胞，计算细胞存活率，实验重复3次。

$
{\rm{存活率}} = \frac{{\rm{给药组吸光度值}}}{{\rm{对照组吸光度值}}} \times 100\% 
$

### 1.8 A549细胞凋亡形态的电子扫描显微镜观察

对数期A549细胞，不同浓度CP-1（200 μg/mL和300 μg/mL）处理24 h，用PBS缓冲液洗涤。按一定方向轻轻摇动。洗涤2次，每次5 min。用2.5%的戊二醛（pH为7.2-7.4）固定液，于4 ℃冰箱内固定1 h。PBS再次清洗样品，浓度依次为30%、50%、70%、80%、90%、100%的乙醇梯度脱水去除样品中水分，每次脱水时间5 min。将样品进行自然干燥，PBS洗3次，电子扫描显微镜观察细胞形态。

### 1.9 单细胞凝胶电泳测定A549细胞DNA损伤

A549细胞用冰冷的PBS洗1次，离心收集，用PBS重悬密度为1×10^6^个/mL；将载玻片的磨砂面向上，40 ℃预热，将预热45 ℃的100 μL的0.5%正常熔点琼脂糖（NMA）铺于载玻片上，盖上干净的盖玻片，再置于4 ℃环境中10 min使NMA凝固。将10 μL细胞（约1×10^4^个）和75 μL的0.7%低溶点琼脂糖LMA（在37 ℃下水浴加热至少20 min使之完全溶化）混合均匀。轻轻揭去盖玻片，迅速将含细胞的LMA滴到第1层琼脂糖上，立即盖上另一干净盖玻片，置4 ℃、10 min使第2层LMA凝固。第2层LMA凝固后，室温下移去盖玻片，滴加预热37 ℃的75 μL的0.7%低溶点琼脂糖LMA，盖上盖玻片4 ℃下凝固。移去盖玻片，将玻片置于平皿中，倒入预冷的细胞裂解液，4 ℃裂解1 h-2 h，取出载玻片用PBS漂洗。将载玻片置于水平电泳槽。倒入新配制的碱性电泳缓冲液（1 mmol/L EDTA，300 mmol/L NaOH），约覆过载玻片胶面0.25 cm左右，室温放置20 min-60 min。在电压25 V条件下，电泳20 min-30 min。电泳后将载玻片置于平皿内。加入0.4 mmol/L Tris-HCl（pH7.5）缓冲液，将载玻片没入，4 ℃中和3次，每次10 min，弃去Tris-HCl缓冲液，每个载玻片加20 μL的PI染液，盖上盖玻片，避光染色10 min。激光共聚焦显微镜543 nm波长的激发光观察。

### 1.10 细胞周期分析

A549细胞接种在六孔板中，不同浓度CP-1（0、100 μg/mL、200 μg/mL和300 μg/mL）处理，培养72 h后，胰酶消化，制成细胞悬液，800 r/min离心5 min，PBS洗两次，4%多聚甲醛固定4 ℃过夜。固定的细胞800 r/min离心8 min，弃上清，PBS洗2遍，加入终浓度为50 U/mL的RNase Inhibitor，37 ℃水浴30 min。加入终浓度为50 μg/mL的碘化丙啶（PI），室温避光40 min，过400目筛，流式细胞仪检测。

### 1.11 总RNA提取及半定量PCR（RT-PCR）

A549细胞培养在培养皿中，分为空白组、CP-1组（200 μg/mL和300 μg/mL）。加CP-1后培养24 h。按总RNA提取试剂盒（Qiagen）说明书提取细胞总RNA，提取的总RNA结果通过1%的琼脂糖凝胶电泳鉴定。按逆转录试剂盒（Fermentas）逆转录得到cDNA。在50 μl的PCR反应体系中，加入2 μL的cDNA、5 μL的反应缓冲液（10×）、2 μL的dNTP及正、反向引物各1.5 μL、5 U的耐热多聚核酸聚合酶（Taq酶）、38 μL的去核酸双蒸水，置于PCR仪中，93 ℃预变性3 min后，进行35个循环（94 ℃变性30 s，58 ℃退火30 s，72 ℃延伸45 s），72 ℃延伸10 min。半胱天冬酶（caspase）-3、caspase-9和β-actin引物序列见表 1。

**1:** 引物列表 List of primers used in polymerase chain reactions

Genes	Forward primer	Reverse primer	Accession
*caspase-3*	5' -TGCTTCTGAGCCATGGTGA-3'	5' -AGTCCAGTTCTGTACCAC-3'	NM_032991
*caspase-9*	5' -GCTCTTCCTTTGTTCATCTCC-3'	5' -CATCTGGCTCGGGGTTACTGC-3'	NM_001229.3
*β-actin*	5' -AAATCTGGCACCACACCTT-3'	5' -AGCACTGTGTTGGCGTAGAG-3'	NG_007992

### 1.12 统计学处理

采用SPSS 17.0软件进行*t*检验，数据采用Mean±SD表示。采用配对样本*t*检验，以*P* < 0.05为差异有统计学意义。

## 2 结果

### 2.1 紫外全波长扫描检测

紫外全波长扫描检测显示，在260 nm、280 nm处没有吸收峰，说明纯化后的CP-1中不含核酸和蛋白质。

### 2.2 薏仁多糖对A549细胞存活率的影响

使用MTT检测CP-1对肺癌细胞A549细胞存活率结果如图 1，CP-1对细胞的抑制作用具有明显的作用时间和作用浓度依赖性。CP-1对A549细胞的存活率影响明显（*P* < 0.05），当CP-1的浓度达到300 μg/mL时，作用72 h，细胞存活率为62.77%。

**1:**
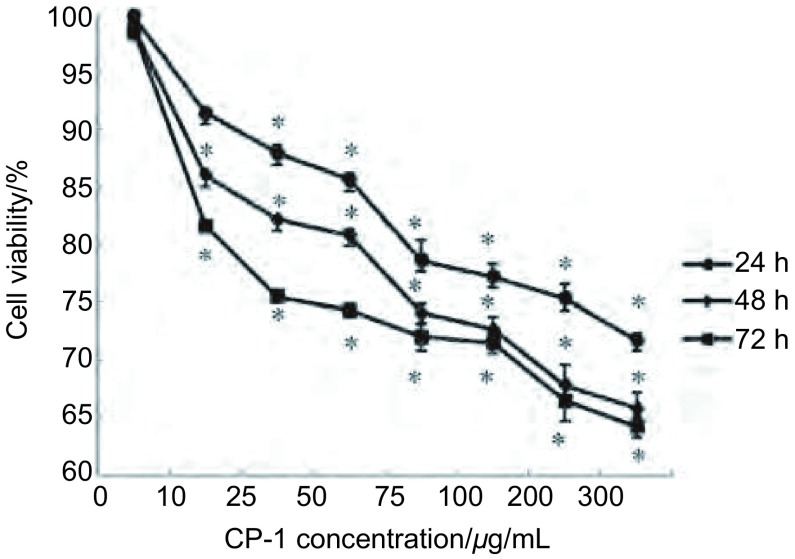
CP-1对A549细胞存活率的影响（*n*=3）（^*^P < 0.05） Effect of CP-1 on the cell viability of A549 cells (*n*=3) (^*^P < 0.05)

### 2.3 A549细胞扫描电子显微镜观察结果

扫描电子显微镜下观察经CP-1处理的A549细胞和空白组细胞。观察结果见图 2。空白组细胞，细胞表面有小的绒毛，并有小的凸起；200 μg/mL CP-1组细胞核细胞凸起开始增加、变大，细胞表面形成褶皱；300 μg/mL CP-1组细胞表面形成大量凸起，并产生凋亡小体。

**2:**
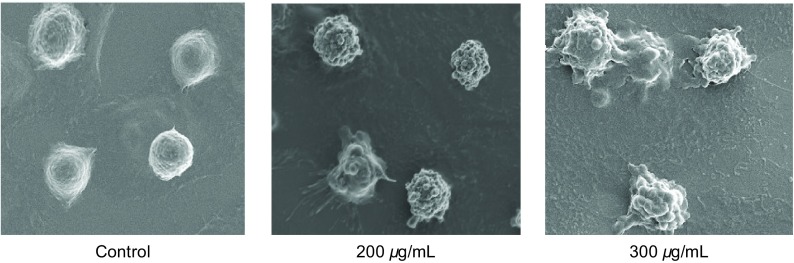
A549细胞SEM观察结果 The morphology of A549 cells observed by SEM

### 2.4 单细胞凝胶电泳（彗星实验）测定A549细胞DNA损伤

A549细胞的单细胞凝胶电泳在激光共聚焦显微镜下观察结果如图 3（x, y: 50 μm），空白组无DNA损伤的细胞表现为一圆形荧光细胞核，200 μg/mL、300 μg/mL组的DNA彗星头很小，头长不超过5 µm，亮度高，慧尾近似椭圆形，在细胞后面形成长的拖尾，呈典型的凋亡彗星尾形状。结果显示CP-1作用后彗星尾距较空白组均增加，差异明显（*P* < 0.05），且二者的改变与作用时间相关。

**3:**
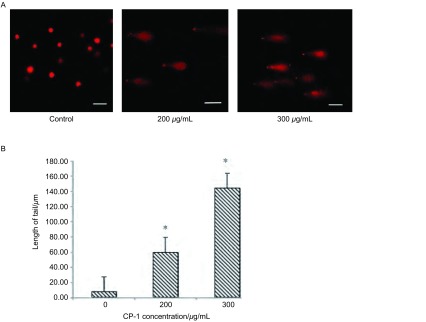
A：A549细胞单细胞凝胶电泳图（比例棒：50 *μ*M）；B：CP-1对慧星尾距计算值的影响（^*^P < 0.05）。 A: Single cell gel electrophoresis of A549 (scale bar: 50 *μ*m); B: The effect of CP-1 in the calculation of comet tail (^*^P < 0.05).

### 2.5 薏仁多糖诱引起S期阻滞

CP-1对A549细胞细胞周期的影响通过流式细胞仪检测，检测结果如图 4。在浓度为300 μg/mL时CP-1造成A549细胞凋亡率较为明显（22.06%）。和空白组相比CP-1形成明显的S期阻滞。

**4:**
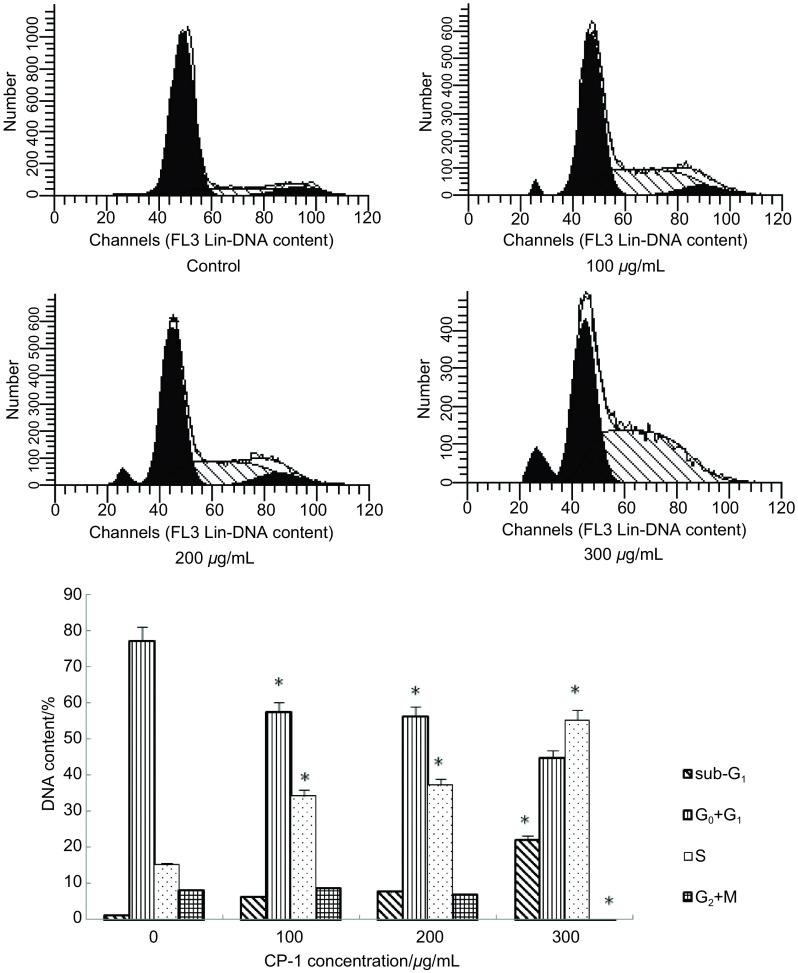
ACP-1对A549细胞周期（DNA含量）的影响（^*^P < 0.05）。 Effect of CP-1 on the cell cycle of A549 cells (^*^P < 0.05).

### 2.6 RT-PCR测定*caspase-3*、*caspase-9*基因表达

不同浓度CP-1作用于A549细胞前后，A549细胞中的*caspase-3*、*caspase-9*基因表达的相对表达量（*caspase-3*/*β-actin*和*caspase-9*/*β-actin*）见图 5A。不同浓度CP-1作用于A549细胞后*caspase-3*、*caspase-9*两种基因的相对表达量明显增加，两种基因表达的差异明显（*P* < 0.05）（图 5B）。

**5:**
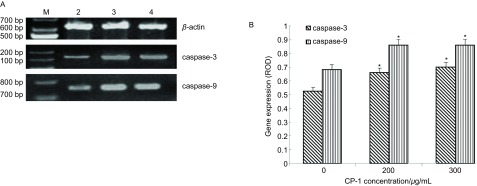
CP-1作用后A549细胞caspase-3和caspase-9的相对表达量。A: RT-PCR的1.2%琼脂糖凝胶电泳；M：Marker; 2：Control; 3：200 *μ*g/mL; 4：300 *μ*g/mL。B：基因相对表达量（^*^P < 0.05）。 The relative quantity of caspase-3 and caspase-9 in A549 cells after CP-1 treatment. A: Electrophoresis of RT-PCR of caspase-3 and caspase-9; M: Marker; 2: Control; 3: 200 *μ*g/mL; 4: 300 *μ*g/mL. B: Relative optical density (ROD) of the gene expression (^*^P < 0.05).

## 3 讨论

肺癌仍然是癌症死亡的主要原因，是当今社会主要的医疗、科学、社会问题^[7]^，目前对于恶性肿瘤的临床治疗手段主要包括放疗、化疗或者手术切除等^[8]^。近年来传统中药的抗癌成分备受人们关注。多糖是植物、真菌、酵母等的主要组成部分，由于多糖的免疫调节机制和抗癌作用，现在多糖的研究引起了越来越多的关注^[9]^。一些多糖具有诱导癌细胞凋亡的作用，其中一些多糖表现出很明显的凋亡作用，但没副作用^[10, 11]^。癌症的产生与发展是由于癌细胞的凋亡功能丧失，使之高增殖与扩散，研究细胞凋亡对于癌症的研究具有很大的意义^[12]^。

在本实验中，我们对CP-1组分诱导A549细胞凋亡的能力进行评估。细胞存活率实验（MTT检测）表明CP-1诱导A549细胞凋亡具有浓度和时间依赖性。在流式细胞仪检测过程中发现凋亡峰，并且造成细胞周期S期阻滞，该作用与一些其它多糖的作用类似，比如枸杞多糖^[13]^。通过扫描电镜我们观察到细胞凋亡前的特征图像，单细胞凝胶电泳实验表明CP-1处理后的彗星尾距拉长也清晰地表明CP-1组分可诱导凋亡。从分子机理研究，caspase家族在细胞凋亡信号传导过程中起核心作用^[14, 15]^。caspase-3是凋亡过程中细胞凋亡的执行者，是多种死亡受体介导的凋亡途径的共同下游效应部分，能够降解细胞骨架蛋白和核蛋白^[16, 17]^。caspase-3活化后，可酶解、切割特异性底物如DNA依赖性蛋白激酶、固醇调控元件结合蛋白等，通过改变其结构或影响特定信号分子而引起细胞凋亡^[18]^。caspase-9属于上游蛋白酶，起到活化caspase-3的作用。RT-PCR显示CP-1使caspase-3和caspase-9表达量提高，表明CP-1通过影响caspase-3和caspase-9表达导致A549的细胞凋亡。

从以上结果分析，CP-1能够降低A549细胞存活率，诱导细胞内的*caspase-3*、*caspase-9*基因相对表达量上调，进而诱导A549细胞产生凋亡小体，S期阻滞，表明薏仁多糖可以诱导人非小细胞肺癌细胞A549细胞凋亡。该研究为以后进一步研究CP-1的抗肿瘤作用提供了有价值的参考。
